# A myriad of factors influencing the implementation of transitional care innovations: a scoping review

**DOI:** 10.1186/s13012-021-01087-2

**Published:** 2021-02-26

**Authors:** Amal Fakha, Lindsay Groenvynck, Bram de Boer, Theo van Achterberg, Jan Hamers, Hilde Verbeek

**Affiliations:** 1 Department of Health Services Research, Faculty of Health, Medicine and Life Sciences, CAPHRI Care and Public Health Research Institute, Maastricht University, Maastricht, The Netherlands; 2 Living Lab in Ageing and Long-Term Care, Maastricht, The Netherlands; 3 Department of Public Health and Primary Care, Academic Centre for Nursing and Midwifery, KU Leuven, Kapucijnenvoer 35, 3000 Leuven, Belgium

**Keywords:** Implementation, Innovation, Care transitions, Transitional care, Long-term care, Factors, Older persons

## Abstract

**Background:**

Care transitions of older persons between multiple care settings are frequently hampered by various issues such as discontinuous care delivery or poor information transfer among healthcare providers. Therefore, several innovations have been developed to optimize transitional care (TC). This review aims to identify which factors influence the implementation of TC innovations.

**Methods:**

As part of TRANS-SENIOR, an international innovative training and research network focusing on enhancing or avoiding care transitions, a scoping review was conducted. The five stages of the Arksey and O’Malley framework were followed. PubMed/MEDLINE, EMBASE, and CINAHL were searched, and eligible studies published between years 2000 and 2020 were retrieved. Data were extracted from the included studies and mapped to the domains and constructs of the Consolidated Framework for Implementation Research (CFIR) and Care Transitions Framework (CTF).

**Results:**

Of 1537 studies identified, 21 were included. Twenty different TC innovations were covered and aimed at improving or preventing transitions between multiple care settings, the majority focused on transitions from hospital to home. Key components of the innovations encompassed transition nurses, teach-back methods, follow-up home visits, partnerships with community services, and transfer units. Twenty-five prominent implementation factors (seven barriers, seven facilitators, and eleven factors with equivalent hindering/facilitating influence) were shown to affect the implementation of TC innovations. Low organizational readiness for implementation and the overall implementation climate were topmost hindering factors. Similarly, failing to target the right population group was commonly reported as a major barrier. Moreover, the presence of skilled users but with restricted knowledge and mixed attitudes about the innovation impeded its implementation. Among the eminent enabling factors, a high-perceived advantage of the innovation by staff, along with encouraging transition roles, and a continuous monitoring process facilitated the implementation of several innovations. Other important factors were a high degree of organizational networks, engaging activities, and culture; these factors had an almost equivalent hindering/facilitating influence.

**Conclusions:**

Addressing the right target population and instituting transition roles in care settings appear to be specific factors to consider during the implementation of TC innovations. Long-term care settings should simultaneously emphasize their organizational readiness for implementation and change, in order to improve transitional care through innovations.

**Supplementary Information:**

The online version contains supplementary material available at 10.1186/s13012-021-01087-2.

Contributions to the literature

- Our study identifies a set of significant factors that influence the implementation of innovations specific to transitional care, which diminishes the existing gap in implementation literature and offers guidance to long-term care organizations in future endeavors for enhancing this type of care for older persons.
- The current findings provide a dynamic and different perspective by addressing the interorganizational aspect of implementing transitional care innovations across multiple long-term care settings.
- The methodology used illustrates the possibility of combining multiple implementation research frameworks to enable a rich and comprehensive study of the influencing factors on implementing transitional care innovations.

## Background

Innovations in transitional care (TC) are often implemented to ensure an optimal continuity of healthcare delivery for older persons who transfer between multiple care settings. Older persons aged 65 years and above are at high risk of adverse events during care transitions due to the prevalence of chronic diseases and multimorbidity [1–7]. Care transitions of older persons are frequently hampered by a diversity of issues, such as, but not limited to, fragmented care, medication errors, or poor communication among healthcare providers [7, 8]. Consequently, the delivery of proper TC for the older population is not always achieved.

There appears to be an urgent need to innovate in order to alleviate the augmented demand for long-term care (LTC) services and promote better and safer care transitions. Based on the World Health Organization’s concept of LTC, we adapted its definition to fit the use throughout this article as “LTC refers to the provision of continuous care activities performed by formal and/or informal/family caregivers to ensure that older persons with or at risk of a significant ongoing loss of intrinsic capacity can maintain a level of functional ability consistent with their basic rights, fundamental freedoms, and human dignity; also it can be achieved through: (a) optimizing the older person’s trajectory of intrinsic capacity, (b) compensating for a loss of capacity by providing the environmental support and care necessary to maintain functional ability at a level that ensures well-being; and can be provided in settings, such as but not limited to: nursing and residential care facilities, assisted living facilities, or private/own home” [9]. To that end, multiple evidence-based TC interventions, models, or programs also referred to as “innovations” have been developed with the goal to improve or prevent transitions between different settings [2]. According to existing literature, we defined the following terms to be used throughout this article: “improve care transitions”—to provide and enhance the transitional care and services delivered during physical relocations of older persons from one care setting to another, with a view to creating optimal benefit as a result of the care transition; “prevent care transitions”—to provide the care and services needed in order to avert an unnecessary or avoidable physical movement of older persons between two care settings or more [2, 5, 7]. The Transitional Care Model and Coleman’s Care Transitions Intervention are examples of interventions designed to improve care transitions from hospital to home [2]. Key components of these interventions include appointing a transition coach or nurse, encouraging patient self-management, and planning hospital discharge [10–12]. While other interventions [13] aim to prevent care transitions from nursing home to hospital through the use of specific advanced care planning tools, alternative interventions focus on providing acute care at home to prevent transitions from home to hospital [14]. The successful implementation of these interventions has been shown to enhance the quality of care, control costs, reduce hospital readmission rates, and ultimately meet patient needs [2, 15]. However, while innovation in TC is encouraged as a solution, its implementation is often difficult and unsuccessful.

The success or failure of the implementation of any innovation within a healthcare setting is usually influenced by multiple factors recognized as either barriers or facilitators [16]. These factors can be linked to either the innovation characteristics, individual professionals, patients and caregivers, organizational structure, or the environmental context [16, 17]. Nevertheless, other factors related to the actual process and activities undertaken to implement an innovation such as the planning, execution, and evaluation methods are as crucial [17]. Similarly, attempts to implement innovations in TC are frequently affected by multiple factors. Among the barriers are limited organizational resources, absence of an implementation climate, complexity of the innovations, and low leadership engagement [18, 19]. Conversely, facilitators include the adaptability of innovations, a high relative advantage of the innovation as perceived by users, and the existence of robust external organizational partnerships [14, 19].

However, to the best of our knowledge, no overview exists on barriers and facilitators that influence the implementation of innovations for preventing or improving care transitions for older persons. Thus, there is a need to explore and map the available evidence on these implementation factors. The main research question of the current study is the following: What are the barriers and facilitators that influence the implementation of TC innovations for older persons in long-term care settings? A secondary question is whether the literature captured the perspectives of older persons and informal or family caregivers on the innovation’s implementation and overall experience, and if so, what was reported as feedback.

## Methods

This scoping review follows the Preferred Reporting Items for Systematic Reviews and Meta-Analyses extension for Scoping Reviews (PRISMA-ScR) checklist [20] (see Additional file 1). The review was conducted according to the five stages described by the Arksey and O’Malley framework [21] and the enhancements proposed by Levac et al. [22].

### Stage 1: identifying the research question

This scoping review is guided by the following question: What are the barriers and facilitators that influence the implementation of TC innovations for older persons in long-term care settings?

### Stage 2: identifying relevant studies

Initially on July 25, 2019, a systematic search of three databases was conducted: PubMed/MEDLINE, EMBASE, and CINAHL; an update was run on March 10, 2020. Four main concept terms were used in the search: implementation; care transition; innovation; and older persons. To formulate the search strings, relevant keywords and synonyms were identified for each concept term in addition to the controlled vocabulary terms (such as MeSH headings in PubMed/MEDLINE). The search strategy was discussed by the authors as well as reviewed by an information specialist. Reference lists of articles that fulfilled the inclusion criteria were searched to identify additional papers. The final search strategy is available (see Additional file 2).

### Stage 3: study selection

Literature published in any language between January 1, 2000, and March 10, 2020, was retrieved.

Original research studies were included. Articles were eligible for inclusion if (a) target population (participants or receiver of care) were all or if the majority were older persons aged 65 years and above (also referred to as patients, older adults, frail older adults, elderly) with long-term care needs and at risk of care transitions; (b) focused on the transfer and physical movement of older persons between two or more care settings with at least one setting providing long-term care; (c) implemented an innovation within a care setting to prevent or improve care transitions; (d) reported on the barriers and facilitators that influenced the implementation process of the innovation; (e) stated the perspectives of the older persons, family, informal caregivers, and/or healthcare providers on the innovation.

After the removal of duplicates, the first author (AF) screened the titles and abstracts for eligibility. In order to increase reliability, the second author (LG) screened a random selection of 10% of the total records for titles and abstracts [23]. Both reviewers then compared their assessment decisions and resolved any differences through discussion and when necessary through consultation with the author (BdB). In the next phase, the two authors (AF, LG) independently screened and discussed 100% of the full texts of those articles deemed eligible [23, 24]. The Preferred Reporting Items for Systematic Reviews and Meta-analyses (PRISMA) flowchart [25] was used to report the study selection process.

### Stage 4: charting the data

#### Development of the data charting forms

A data charting form consisting of two parts was developed. *Data charting form*—*part 1* comprised the following: title; authors; year; country; study aim; design and methodology; population; setting; innovation description; duration and phase of implementation; presence of barriers and/or facilitators to innovation implementation; reported themes of barriers and/or facilitators to the implementation of the innovation; perspectives of older persons, family, or informal caregivers and/or providers on the innovation; and reported implications of the innovation. *Data charting form—part 2* was devised to map barriers and facilitators as identified in the studies to the Consolidated Framework for Implementation Research (CFIR) [26] and the Care Transitions Framework (CTF) [27].

The CFIR is composed of five domains: (i) *intervention characteristics*; (ii) *outer setting*; (iii) *inner setting*; (iv) *characteristics of individuals*; (v) *process*, and 39 standardized constructs and subconstructs [26]. This framework helps researchers identify the factors (i.e., barriers and facilitators) that influence the implementation of innovations [28]. Moreover, specific constructs from the CTF [27] were selected and used in supplement to the CFIR (see Additional file 3). The CTF is an adaptation of the CFIR, whereby it incorporates all the CFIR constructs in addition to new ones, which are mostly relevant to transitional care.

#### Testing of data charting forms and the charting process

Both forms were tested initially on two articles, and then results were discussed critically within the research team. It was agreed to include additional elements to describe further the innovations’ features in data charting form 1. In the data charting, the implementation factors and themes were extracted from the included articles and then mapped to the CFIR’s relevant domains, constructs, and the selected CTF constructs using the CFIR codebook [29]. Subsequently, the CFIR rating rules were used to determine each factor’s influence as negative: a barrier, or positive: a facilitator [30]. Two authors (AF, TvA) charted data independently from five randomly selected articles. Disagreements on mapping factors to CFIR/CTF constructs were resolved between the two authors leading to a consensus. Afterwards, author AF completed the full data charting for all the included articles.

### Stage 5: collating, summarizing, and reporting the results

The data charted were synthesized as follows:

- Description of included studies: classification of the studies into four groups according to the care transition pathways of each TC innovation; included the author(s), year of publication, country, objective, population, design, and methods.
- Description of the TC innovations: classification of the innovations into four groups according to the specific care transition pathways; included the target population, key components, and the CFIR domains influencing their implementation.
- Barriers and facilitators to implementation of TC innovations: the frequency of the reported factors identified as barriers and/or facilitators to the implementation was calculated based on their presence in the number of studies.
- Perspectives of older persons, family, or informal caregivers: a narrative description of the feedback on the overall experience, satisfaction with, or views on the implementation of the TC innovation.

## Results

### Study selection

**Fig. 1:**
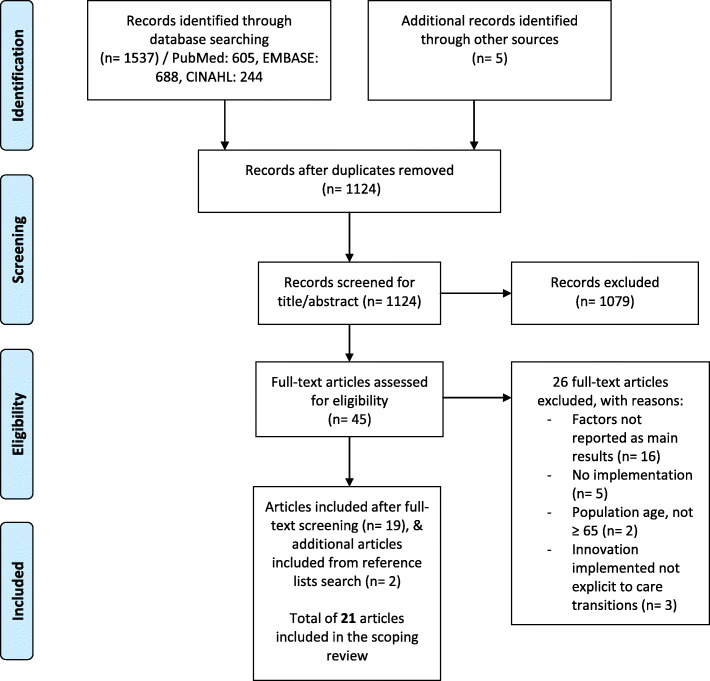
PRISMA flowchart of study selection process

Initially, 1537 studies were identified, and 21 were included in the final stage. The flowchart for the selection process is depicted in Fig. 1.

### Study characteristics

The 21 studies included described the implementation of 20 different TC innovations (see Table 1). Almost half of the studies (*N* = 11, 52%) originated from the USA, and five were from Europe. The majority of the studies were process evaluations and were performed during or post the implementation of a TC innovation to examine the influencing factors. Most studies used qualitative research methods, and 11 utilized a preselected evaluation, implementation, or quality-related framework, tool, model, or instrument to guide data collection such as interviews and/or data analysis.

**Table 1:** Characteristics of the 21 included studies

Author(s), year of publication, country	Objective and timing of data collection	TC innovation name	Study population, total sample (*n*)	Design and methods
Group I—studies focused on TC innovations to *improve* care transitions from *hospital to home*				
Bradway et al. 2012, USA [31]	To describe the barriers and facilitators to implementing a transitional care intervention for cognitively impaired older adults and their caregivers led by advanced practice nurses ➢ Post-implementation	APN-directed TCM: Advanced Practice Nurse-Directed Transitional Care Model	Healthcare professionals (*n* = 3): ▪ Advanced practice nurses	Exploratory qualitative: ▪ Case summaries for patient-caregiver dyads completed by APNs at end of intervention—15 randomly selected ▪ Field notes taken by study investigators during biweekly APNs’ case conferences
Couture et al. 2016, Canada [18]	To evaluate and explain the barriers and facilitators to the implementation of a pilot intervention—introducing the role of transitional care managers within a public healthcare system ➢ During implementation	TCM Role: Transitional Care Manager Role	Healthcare professionals (*n* = 29): ▪ Transitional care managers ▪ Hospital workers ▪ Social workers ▪ Staff of health and social service centers	Process evaluation: ▪ Assessed the fidelity, acceptability, and appropriateness (contextual factors) ▪ Focus groups ▪ Direct observations ▪ TCMs’ activity grids and logbooks ▪ Meeting minutes and documents of coordinating and implementation committee
Hung et al. 2015, USA [32]	To examine and describe the successes and challenges of the implementation of a pilot community-based transitional care program ➢ Post-implementation	Community-based TCP: Community-based Transitional Care Program	Healthcare professionals and program’s management team (*n* = 7): ▪ Interprofessional team of program staff (nurses, social workers) ▪ Members of program’s steering committee	Qualitative: ▪ Semi-structured interviews guided by the Organizational Readiness to Change Assessment instrument ▪ Analysis using PARIHS framework (contextual factors, evidence, facilitation techniques)
Hung et al. 2018, USA [33]	To examine the key contextual features enabling the implementation and hospital-wide scaling of a community-based transitional care program ➢ Post-implementation	Community-based TCP: Community-based Transitional Care Program	Healthcare professionals and program’s management team (*n* = 17): ▪ Program director and manager ▪ Program staff (nurses, social workers, wellness coaches) ▪ Members of program’s steering committee	Qualitative: ▪ Semi-structured interviews guided by the Organizational Readiness to Change Assessment instrument ▪ Analysis using Care Transitions Framework (domains—intervention, organizational, and patient characteristics, implementation process, measures, and outcomes)
McNeil et al. 2016, Canada [34]	To evaluate the effectiveness and identify barriers to and facilitators of the implementation of an intervention involving patient handoff between a hospital-based care transitions nurse and a community-based response nurse ➢ Post-implementation	CTI-Handoff: Care Transition Intervention Handoff	▪ Care transitions and rapid response nurses (*n* = not specified) ▪ Managers and executives (*n* = 4)	Qualitative: ▪ Focus group discussions ▪ Individual interviews
Naylor et al. 2009, USA [35]	To identify the major facilitators and barriers to implementing the Transitional Care Model in an insurance organization over the phases of start-up and roll-out ➢ Pre- and during implementation	TCM: Transitional Care Model	Healthcare professionals and project team (*n* = 19): ▪ Implementation staff ▪ Case managers ▪ Transitional care nurses ▪ Senior leaders and managers	Qualitative: ▪ Semi-structured interviews guided by Everett Roger’s framework for diffusion of innovations (focus on: staff involvement, culture, communication channels, model integration within organization, ease/difficulty of start-up phase)
Nurjono et al. 2019, Singapore [36]	To evaluate the implementation fidelity of a transitional care program ➢ Post-implementation	NUHS-RHS TCP: National University Health System-Regional Health Systems Transitional Care Program	Healthcare professionals (*n* = 25): ▪ Care coordinators, program managers, physicians ○ Family caregivers (*n* = 45)	Realist evaluation: ▪ Using the Conceptual Framework of Implementation Fidelity (moderating factors: context, participant responsiveness, program complexity, facilitating strategies, recruitment) ▪ Interviews ▪ Observations ▪ Reviews of medical records and program databases
Parrish et al. 2009, USA [37]	To identify factors that promote the sustainability of the implementation of a care transition intervention pilot ➢ Post-implementation	CTI: Care Transitions Intervention	▪ Pilot project team members for 10 sites (*n* = 30–40)^a^, including transition coaches (*n* = at least 10)^a^ ○ Patients (*n* = 791, out of which 69.4% are aged 66+)	Mixed methods: ▪ Surveys ▪ Interviews ▪ Final project narrative reports, data reports ▪ Comparison of pre- and post-project sustainability plans ▪ 5 variables used to assess sustainability factors (leadership, transition coaches staff, project management, team commitment, sustainability plan) ▪ Care Transition Measure (CTM) to assess quality of care transition ▪ Patient Activation Assessment (PAA) to assess level of patient activation in the 4 pillars of the CTI
Rosstad et al. 2015, Norway [38]	To investigate the implementation process of a care pathway for elderly patients into the daily practice of healthcare professionals ➢ During and post-implementation	PaTH: Patient Trajectory for Home-dwelling elders	Healthcare professionals (*n* = 60): ▪ Home care managers ▪ Home care head nurses ▪ Nurses ▪ Nursing assistants	Comparative qualitative process evaluation: ▪ Semi-structured interviews guided by questions focused on staff involvement and expectations, care pathway introduction and efforts to use it, challenges, promoting factors, benefits, sustainability ▪ Focus group discussions ▪ Field notes (by study investigator on overall implementation process) ▪ Meeting minutes (conference calls with head nurses and home care managers performed by study investigator) ▪ Analysis guided by the Normalization Process Theory core constructs (coherence, cognitive participation, collective action, reflexive monitoring)
Williams et al. 2014, USA [39]	To examine the successes and failures experienced by implementing Project BOOST aiming to enhance transition from hospital to home ➢ During implementation in cohorts and post-implementation in pilot sites	BOOST: Better Outcomes by Optimizing Safe Transitions	▪ Local team leaders of (*n* = 6 pilot hospital sites) ▪ Hospital team leaders of (*n* = 27 cohort sites)	Evaluation: ▪ Telephone interviews guided by basic implementation questions and opinions on intervention elements in pilot sites ▪ Survey with hospital cohorts on implementation occurrence, when, and how
Xiang et al. 2018, USA [40]	To examine the experiences of community-based organizations implementing the Bridge Model of Transitional Care, and identify facilitators and barriers associated with the implementation and sustainability of the model ➢ During and post-implementation	The Bridge Model	Healthcare professionals (*n* = 13): ▪ Clinical supervisors ▪ Program coordinators	Qualitative case study: ▪ Semi-structured interviews by telephone, guided by 3 domains of successful implementation of the PARIHS framework (evidence, context, leadership, evaluation, facilitation)
Group II—studies focused on TC innovations to *improve* care transitions from *hospital to intermediary care places* (*residential care or rehabilitation facility*) *to a final destination*				
Everink et al. 2017, the Netherlands [41]	To evaluate the feasibility of implementing an Integrated Care Pathway in Geriatric Rehabilitation ➢ Post-implementation	ICP Geri-Rehab: Integrated Care Pathway in Geriatric Rehabilitation for People with Complex Health Problems	Healthcare professionals (*n* = 19): ▪ Elderly care physicians ▪ Nurses (specialized, discharge) ▪ Physiotherapists ▪ Professionals from home care organizations ○ Informal caregivers (*n* = 37) ○ Patients (*n* = 113)	Process evaluation: ▪ Using Saunders and colleagues framework (fidelity, dose delivered, satisfaction, contextual and external factors) ▪ Semi-structured group interviews ▪ Face-to-face interviews ▪ Self-administered questionnaires ▪ Patient files ▪ Meeting minutes
Masters et al. 2008, Australia [42]	To examine reports from providers to reveal enablers and barriers to compliance with the key requirements of a transition care program ➢ Post-implementation	TC Places: Transition Care Places	▪ Organizations providing transition care services (*n* = 23 organizations)	Qualitative: ▪ Content analysis of quality self-reports
Plochg et al. 2005, the Netherlands [43]	To assess the functioning and implementation of an intermediate care model between a hospital and a residential home ➢ Post-implementation	ICM: Intermediate Care Model	Healthcare professionals and management staff from the residential home and medical center (*n* = 21): ▪ Leadership (general manager, director, head of care department, chair board of directors, chair of medical specialists) ▪ Physicians ▪ Nurses (liaison, geriatric nurse specialist, liaison nurse-head of discharge unit, registered nurse-head of transfer unit, nursing assistant of transfer unit) ▪ Occupational/physical therapists	Process evaluation: ▪ Semi-structured interviews ▪ Analysis using a typology of quality systems based on 5 elements (structural assets, allocation of responsibilities, protocols, information transfer, and monitoring/feedback cycles), and Grol’s model on effective implementation
Renehan et al. 2013, Australia [44]	To evaluate the implementation and effectiveness of a Transition Care Cognitive Assessment and Management Pilot ➢ Post-implementation	TC CAMP: Transition Care Cognitive Assessment and Management Pilot	Healthcare professionals (*n* = 17): ▪ TC CAMP facility and health services staff (nursing, management, allied health, team leaders, therapists, clinical nurse consultant) ▪ Unit managers of final destination facility ○ Family caregivers (*n* = 7)	Process and outcome evaluation: ▪ Structured interviews ▪ Focus group discussions ▪ Medical records file audits
Group III—studies focused on TC innovations to *improve* care transitions from *hospital or home to nursing/residential care facility*				
Sutton et al. 2016, UK [45]	To characterize the challenges experienced in a quality improvement project aiming to improve transitions for older people ➢Post-implementation	QIP-TC: Quality Improvement Project to Improve Transitions of Care for Older People	Healthcare professionals and project team (*n* = 12): ▪ Care home staff (managers, owners, assistants, frontline) ▪ Hospital-based staff ▪ Project staff	Ethnographic process evaluation: ▪ Observations ▪ Semi-structured interviews ▪ Project documents (progress reports, meeting minutes)
Van Mierlo et al. 2015, the Netherlands [46]	To evaluate a mental healthcare transfer intervention after the movement of a person with dementia into a nursing home, and to investigate factors that influence its successful implementation ➢ Post-implementation	CPN: Follow-up visit by Community Psychiatric Nurse	Healthcare professionals (*n* = 28): ▪ Professional nursing home carers ▪ Community psychiatric nurses ▪ Nursing home managers ▪ Outpatient clinic managers ▪ General practitioners ▪ Team manager—center for people with dementia ▪ Healthcare insurer ○ Family caregivers (*n* = 5)	Evaluation: ▪ Semi-structured interviews based on the Theoretical Model of Adaptive Implementation (external factors, different phases and levels of implementation process)
Group IV—studies focused on TC innovations to *prevent* care transitions from *nursing facility or home to hospital*				
Brody et al. 2019, USA [14]	To examine the barriers to and facilitators of the implementation of Hospital at Home Plus 30 days of transitional care program during its first year of operation ➢ During implementation	HaH Plus program: Hospital at Home Plus Program	Healthcare professionals (*n* = 27): ▪ Team physicians ▪ Nurse practitioners/leaders ▪ Social workers ▪ Leadership staff ▪ Executives ▪ Home health agency staff	Qualitative: ▪ Primers to help recall of key events/factors and develop interview guide ▪ Semi-structured interviews ▪ Focus group discussions
Ersek et al. 2018, USA [47]	To explore the stakeholders’ perspectives on the implementation of OPTIMISTIC program, which aims to reduce hospitalizations from nursing facility; specifically the program’s effective components, facilitating adoption features, and barriers to implementation ➢ During implementation	OPTIMISTIC: Optimizing Patient Transfers, Impacting Medical Quality, and Improving Symptoms: Transforming Institutional Care project	Healthcare professionals (*n* = 53): ▪ Primary care providers ▪ Nursing home staff and leadership ▪ OPTIMISTIC clinical staff ○ Family members of nursing home residents (*n* = 10)	Evaluation: ▪ Using Stetler framework of formative evaluation ▪ Semi-structured group and individual interviews
Hirschman et al. 2017, USA [48]	To describe the experiences of healthcare providers involved in adapting and testing the feasibility of implementing a care innovation by combining two models: the patient-centered medical home and the transitional care model ➢ Post-implementation	PCMH + TCM: Patient-Centered Medical Home + Transitional Care Model	▪ Transitional care nurses (*n* = 2) ▪ Clinicians (*n* = 2–4/site^a^, 5 sites)	Qualitative: ▪ Surveys (open-ended questions)
Rask et al. 2017, USA [49]	To identify contextual and implementation factors impacting the effectiveness of an organizational-level intervention to reduce preventable hospital readmissions from affiliated skilled nursing facilities (SNFs) ➢ Post-implementation	INTERACT II: Interventions to Reduce Acute Care Transfers	▪ Quality improvement organization staff (*n* = 4)^a^ ▪ Leaders and nurses of SNF corporations (*n* = 6)^a^ ▪ SNF staff (*n* = 2–3/facility)^a^	Evaluation: ▪ Interviews with open-ended questions based on contextual factors’ domains of the Model for Understanding Success in Quality tool (external environment, organization, QI support and capacity, microsystem, miscellaneous)

^a^Estimations

Study populations across all studies were comprised of multiple healthcare professionals and providers. Only six studies included older persons or family/informal caregivers and explored their perspectives on the TC innovations [36, 37, 41, 44, 46, 47].

### Key features of the TC innovations

Sixteen innovations focused on improving care transitions for older persons, while four focused on preventing transitions. TC innovations were classified into groups according to the care transition pathways (see Table 2).

**Table 2:** Description and key features of the 20 TC innovations

TC innovation name	Target population	TC innovation—aims and key components	CFIR domains associated with the TC innovation’s implementation
Group I: 10 TC innovations to *improve* care transitions^b^ from *hospital to home*			
APN-directed TCM: Advanced Practice Nurse-directed Transitional Care Model—Bradway et al. 2012, USA [31]	• ≥ 65 years older adults, hospitalized, cognitively impaired • Presence of a family caregiver (CG)	➢ Aims: to improve patient outcomes and ensure a safe and timely transition • Advanced practice nurse, role: ○ daily hospital visits to patient-CG dyad ○ home (or SNF) visits^a^ within 24 h post-discharge, a minimum of 4 ○ telephone follow-up and support ○ development of individualized care plans, patient-CG goals ○ implementation of risk reduction strategies to minimize effects of cognitive impairment ○ coordination with a multidisciplinary local team of healthcare experts ○ building CG ability to identify early symptoms and apply strategies to prevent poor outcomes	✓ Intervention characteristics ✓ Outer setting ✓ Characteristics of individuals ✓ Process
TCM Role: Transitional Care Manager Role—Couture et al. 2016, Canada [18]	• ≥ 70 years older adults, and/or chronically ill • Being discharged from hospital, or end of acute care is predictable	➢ Aims: to improve existing discharge planning practices • Transitional care manager (social worker, or any other healthcare professional, except nurses), a liaison agent role: ○ improvement of discharge planning by management of environmental and community barriers ○ exchange of patient information between providers ○ coordination of care and problem-solving of transitional care	✓ Intervention characteristics ✓ Inner setting
Community-based TCP: Community-based Transitional Care Program—Hung et al. 2015/2018, USA [32, 33]	• ≥ 65 years older adults • About to be discharged from hospital • At high risk of readmission	➢ Aims: to reduce preventable hospital readmissions and improve patient’s quality of life at home and in the community • Health coach (nurse or social worker), role: ○ home visits (within 24–48 h) post-discharge, follow-up phone calls and appointments with primary care providers • Discharge planning using “teach-back” methods • Connecting older adults to community services and resources • Support system network • Advanced care planning • Wellness coach up to 6 months	✓ Intervention characteristics ✓ Outer setting ✓ Inner setting ✓ Characteristics of individuals ✓ Process
CTI-Handoff: Care Transition Intervention Handoff—McNeil et al. 2016, Canada [34]	• Frail older adults with complex conditions • Discharged from hospital and require home care	➢ Aims: to reduce readmissions, improve information transfer, and enhance patient satisfaction • Patient care handoff between hospital care transition nurse and community rapid response nurse • Home care and follow-up period up to 30 days • Referral to hospital-based chronic disease management clinics	✓ Intervention characteristics ✓ Inner setting ✓ Characteristics of individuals ✓ Process
TCM: Transitional Care Model—Naylor et al. 2009, USA [35]	• Chronically ill, high-risk older adults • Hospitalized with multiple chronic conditions	➢ Aims: to improve patient outcomes, reduce readmissions, and reduce healthcare costs • Transitional care nurse, role: ○ primary care coordinator among providers and ensuring a multidisciplinary approach with open communication ○ in-hospital patient case assessment and development of care plan ○ regular home visits and ongoing telephone support (7 days/week over 2 months post-discharge) ○ continuity of medical care with hospital/primary care and accompanying patients on follow-up visits • Early identification and response to health risks • Active engagement of patients and their family/informal caregivers by focusing on education and support	✓ Intervention characteristics ✓ Outer setting ✓ Inner setting ✓ Characteristics of individuals ✓ Process
NUHS-RHS TCP: National University Health System-Regional Health Systems Transitional Care Program—Nurjono et al. 2019, Singapore [36]	• Older adults, and/or with complex healthcare needs • Frequent admitters to hospital • Have limited ambulation and caregivers at home	➢ Aims: to improve quality of care, reduce hospital utilizations, and reduce healthcare related costs • Care coordinator, an integrator role: ○ home visits, telephone monitoring ○ needs and home environment assessment ○ development of personalized care ○ promotion of self-care • Care coordination with a network of medical and social care providers in/out of hospital	✓ Intervention characteristics ✓ Outer setting ✓ Inner setting ✓ Characteristics of individuals ✓ Process
CTI: Care Transitions Intervention—Parrish et al. 2009, USA [37]	• Older adults, in hospital for chronic disease, and requiring long-term care	➢ Aims: to enhance patient safety during transitions • 4-week intervention • Transition coach (nurse or social worker), role: ○ hospital visit ○ 1 home visit (24–72 h post-discharge) ○ 3 follow-up phone calls • Improvement of patient’s capacity: ○ medication self-management ○ using a patient-centered health record ○ knowledge of “red flags” ○ making primary care provider/specialist appointments	✓ Inner setting ✓ Process
PaTH: Patient Trajectory for Home-dwelling elders—Rosstad et al. 2015, Norway [38]	• Elderly patients requiring home care services after discharge from the hospital	➢ Aims: to improve continuity of care and reduce the need of institutional care • Continuity of care from hospital and follow-up of home care recipients • Exchange of patient discharge information between the hospital, local healthcare allocations (municipality-level), and home care services: ○ local healthcare allocations office evaluate and decide on care assistance ○ home care service prepares for transition ○ home care nurse performs comprehensive patient assessment within 3 days upon discharge ○ general practitioner consults patient 14 days post-discharge ○ district nurse/nursing assistant performs extended assessment during the first 4 weeks • Communication among services through a patient daily care plan and patient checklist document	✓ Intervention characteristics ✓ Inner setting ✓ Characteristics of individuals ✓ Process
BOOST: Better Outcomes by Optimizing Safe Transitions—Williams et al. 2014, USA [39]	• Older adults • At high-risk of adverse events post-hospital discharge	➢ Aims: to improve patient’s discharge and reduce errors, reduce 30-day readmission rates, and improve patient satisfaction • Comprehensive intervention toolkit for clinical teams: ○ risk assessment ○ patient/caregiver education tools ○ teach back ○ discharge summary ○ follow-up phone call within 72 h • Implementation guide for multidisciplinary teams • Individual physician mentoring • BOOST collaborative across hospitals	✓ Intervention characteristics ✓ Inner setting ✓ Process
The Bridge Model—Xiang et al. 2018, USA [40]	• Older adults with complex care needs • Discharged from an inpatient hospital stay • At risk of readmission due to psychosocial determinants	➢ Aims: to improve care transition and prevent readmission by addressing the psychosocial determinants • Bridge care coordinator (social worker), role: ○ hospital visits ○ biopsychosocial needs assessment and development of a care plan ○ care coordination and follow-up in person or by telephone throughout 30 days post-discharge • Collaboration of hospital and community-based organizations for aging services	✓ Intervention characteristics ✓ Outer setting ✓ Inner setting ✓ Process
Group II: 4 TC innovations to *improve* care transitions^b^ from *hospital to intermediary care places* (*residential care or rehabilitation facility*) *to a final destination*			
ICP Geri-Rehab: Integrated Care Pathway in Geriatric Rehabilitation for People with Complex Health Problems—Everink et al. 2017, the Netherlands [41]	• ≥ 65 years frail older adults with complex health problems • Previously admitted to hospital and geriatric rehabilitation care	➢ Aims: to improve communication between healthcare providers and enhance the triage process during transitions • Triage instrument for intermediary geriatric rehabilitation facility: ○ assessment of patient need for admission before movement to home setting • Care pathway coordinator, role: ○ communication between professionals and across settings ○ coordination and continuity of care • Active involvement of patients and informal caregivers • Patient discharge summaries • Evaluation meetings and open communication across providers	✓ Intervention characteristics ✓ Outer setting ✓ Inner setting ✓ Process
TC Places: Transition Care Places—Masters et al. 2008, Australia [42]	• Older adults • Concluded an acute hospital episode • Requiring more time and support in a non-acute setting to complete their restorative process and optimize their functional capacity	➢ Aims: to minimize inappropriate extended hospital length of stay, prevent inappropriate admission to residential aged care, and optimize patient’s independence/functional capacity • TC intermediary places located in a residential care facility or a community setting • Delivery of transition care in TC places: ○ goal-orientated, individualized ○ time-limited care ○ low-intensity therapies and services ○ case management • Finalization of long-term care arrangements	✓ Outer setting ✓ Inner setting ✓ Process
ICM: Intermediate Care Model—Plochg et al. 2005, the Netherlands [43]	• Frail older adults, chronically ill • Completed medical treatment at hospital but unfit to go home • Require long-term care	➢ Aims: to reduce length of hospital stays, prevent hospital readmissions, retain patient’s independence • Transfer unit (beds) located in a residential home: ○ low-intensity early discharge care model ○ provision of services bridging the acute, primary, and social care • Coordination of transitions by hospital liaison nurse	✓ Intervention characteristics ✓ Inner setting ✓ Characteristics of individuals ✓ Process
TC CAMP: Transition Care Cognitive Assessment and Management Pilot—Renehan et al. 2013, Australia [44]	• ≥ 65 years older adults • With cognitive impairment (dementia) • At conclusion of an episode of hospital care	➢ Aims: to reduce readmissions • TC CAMP intermediary restorative care places located in a residential care facility • Clinical nurse consultant (CNC), role: ○ case management ○ individualized care plan ○ behavioral management • “Key to Me,” patient information tool	✓ Intervention characteristics ✓ Inner setting ✓ Characteristics of individuals ✓ Process
Group III: 2 TC innovations to *improve* care transitions^b^ from *hospital or home to nursing/residential care facility*			
QIP-TC: Quality Improvement Project to Improve Transitions of Care for Older People—Sutton et al. 2016, UK [45]	• Older people • In transition between hospital and residential care settings during period of acute illness	➢ Aims: to improve communication and information transfer and reduce readmissions • Community geriatric service: ○ geriatrician and community nurse ○ 24-h telephone support and advisory service to facility staff • Patient information summary form	✓ Intervention characteristics ✓ Outer setting ✓ Characteristics of individuals ✓ Process
CPN: Follow-up visit by Community Psychiatric Nurse—Van Mierlo et al. 2015, the Netherlands [46]	• Older people with dementia behavioral disturbances • Expected to be admitted or are advised to move from home into a nursing facility	➢ Aims: to promote continuity of care and improve quality of care • Community psychiatric nurse (CPN), role: ○ follow-up visit 6 weeks after placement in a nursing home ○ clinical and behavioral assessment ○ support and advice to facility nurse ○ support to family caregiver	✓ Intervention characteristics ✓ Outer setting ✓ Inner setting ✓ Characteristics of individuals ✓ Process
Group IV: 4 TC innovations to *prevent* care transitions^c^ from *nursing facility or home to hospital*			
HaH Plus program: Hospital at Home Plus Program—Brody et al. 2019, USA [14]	• ≥ 65 years older adults, requiring inpatient admission	➢ Aims: to reduce mortality, readmission rates, costs, and achieve better patient/caregiver satisfaction • Acute-level care services provision at home as a substitute for hospital admission, *plus* • A 30-day post-acute period of transitional care bundle (self-management, care coordination)	✓ Intervention characteristics ✓ Outer setting ✓ Inner setting ✓ Characteristics of individuals ✓ Process
OPTIMISTIC: Optimizing Patient Transfers, Impacting Medical Quality, and Improving Symptoms: Transforming Institutional Care project—Ersek et al. 2018, USA [47]	• Frail older residents of nursing facility	➢ Aims: to reduce hospitalizations • OPTIMISTIC RNs’ and NPs’ role: ○ identification, assessment, and management of acute conditions in nursing home ○ promotion of INTERACT (Interventions to Reduce Acute Care Transfers) tools usage • Care activities organized within 3 care cores: medical, transitions, palliative	✓ Intervention characteristics ✓ Inner setting ✓ Characteristics of individuals ✓ Process
PCMH + TCM: Patient-Centered Medical Home + Transitional Care Model—Hirschman et al. 2017, USA [48]	• ≥ 65 years older adults with multiple chronic conditions • In community settings	➢ Aims: to prevent avoidable emergency room visits and hospitalizations, and provide a continuous care management • Patient-centered holistic approach • Combination of disease management in primary care settings and home care: ○ coordination of care during an episode of acute illness across settings, facilitated by: • Transitional care nurse (TCN), role: ○ home visits, telephone support ○ active engagement of patient, family caregivers, and collaboration with primary care providers ○ coordination of education and community services to develop self-management skills	✓ Outer setting ✓ Inner setting ✓ Characteristics of individuals ✓ Process
INTERACT II: Interventions to Reduce Acute Care Transfers—Rask et al. 2017, USA [49]	• Residents of long-term care settings	➢ Aims: to reduce the frequency of transfers to hospital, and improve quality of care for residents • Identification, evaluation, and communication of resident status changes • Use of 4 practice tools: ○ quality improvement ○ communication ○ decision support ○ advance care planning	✓ Outer setting ✓ Inner setting ✓ Characteristics of individuals ✓ Process

*RNs* registered nurses, *NPs* nurse practitioners

^a^Sometimes patients are admitted to (SNF) skilled nursing facility prior to going home; they receive visits in both settings

^b^“Improve care transitions”—to provide and enhance the transitional care and services delivered during physical relocations of older persons from one care setting to another, with a view to creating optimal benefit as a result of the care transition

^c^“Prevent care transitions”—to provide the care and services needed in order to avert an unnecessary or avoidable physical movement of older persons between two care settings or more

### Description of the four groups of TC innovations

*Care transitions from hospital to home settings* were the focus of ten TC innovations. Improving care transitions was the main aim of these innovations with common goals to reduce hospital readmissions, lower healthcare costs [31, 34–36, 39, 40], enhance older persons’ quality of life [18, 32, 33] and satisfaction [34, 39], and scale down the need for institutional care [38]. Mostly, these innovations targeted older persons with chronic and complex conditions discharged from hospital, requiring long-term care at home, and who were at higher risk of readmission.

The common component across the innovations was the existence of a healthcare professional with a “transition role,” such as a transition nurse, health coach, care coordinator, social worker, or community nurse. The role served to ameliorate the transition journey from hospital to home by primarily providing follow-up, developing individualized care plans, and coordinating care.

*Care transitions from hospital to intermediary care places to a final destination* were the focus of four TC innovations. These innovations aimed to improve care transitions with common objectives, such as reducing the length of hospital stays, relieving hospital bed-blocking, and preventing inappropriate admission to residential aged care [42, 43]. All four innovations were designed for older persons who concluded an episode of acute care at hospital but were unfit to transfer to home or another final long-term care destination. The creation of “transition intermediary care places” such as transfer beds hosted within a residential care facility or community setting was the notable component across these innovations [41–44]. Hence, the four TC innovations allowed extra time to organize a more personalized arrangement for the long-term care final destination for older persons.

*Care transitions from hospital or home to nursing/residential care facility* were the focus of two TC innovations. The goal of these innovations was to improve care transitions with the objective to enhance information transfer between hospitals and nursing facilities and promote continuity of care. The essential aspect of both innovations was the provision of “transition advice & support” to nursing facility staff. This was enabled through the arrangement of community geriatric services and a psychiatric community nurse [45, 46].

*Care transitions from nursing facility or home to hospital* were the focus of four TC innovations. These innovations aimed to prevent care transitions. Hence, the main objectives were the provision of a value-based and patient-centered high-quality care [14], as well as the reduction and prevention of avoidable hospitalizations [47, 48], and reducing the frequency of transfers to acute hospital care [49]. The unique component of all four innovations was “transition care management in place.”

### Barriers and facilitators to the implementation of TC innovations

**Fig. 2:**
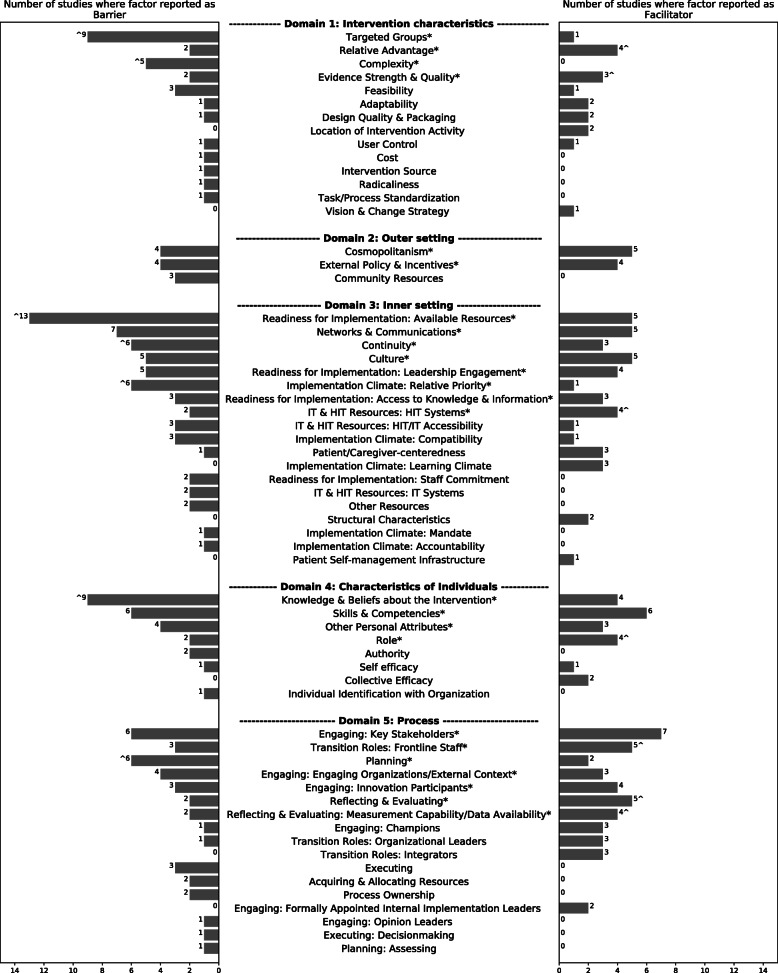
Frequency of reported barriers and facilitators to TC innovations implementation, mapped to CFIR&CTF (61 constructs). The asterisk(*) represents factors cited by at least 5 studies (25%) as a barrier and/or facilitator; the caret(^) denotes factor as a predominant barrier or facilitator; total number of studies is 21

Factors reported in the 21 studies could be mapped to 61 CFIR&CTF constructs, out of which 19 were reported as barriers only, 8 as facilitators only, and 34 as both barriers and facilitators. Among these 34 factors, 15 were reported as having both influences concurrently in the same study. The reporting frequency, presented as number of studies, for the barriers and facilitators influencing the implementation of the transitional care innovations as mapped to the CFIR&CTF constructs is shown in Fig. 2.

**Fig. 3:**
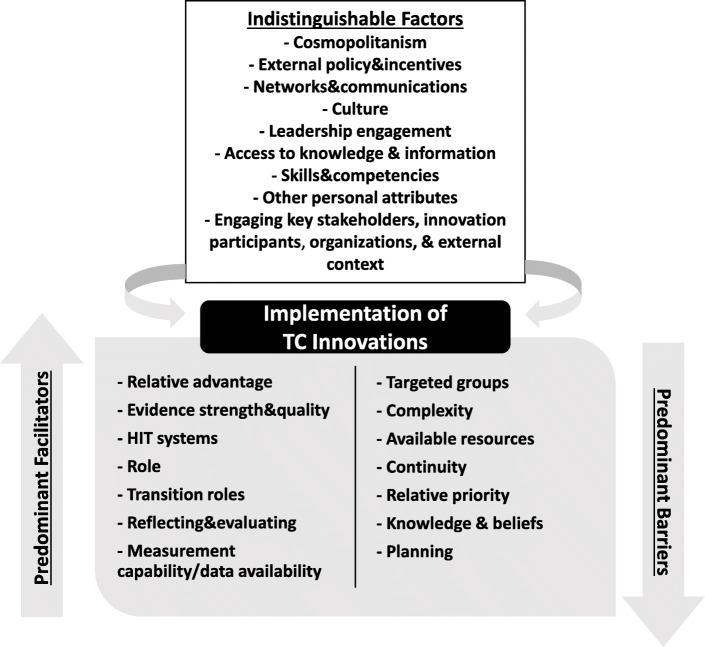
Overview of the factors influencing the implementation of TC innovations

The most commonly reported domains impacting implementation were process (20 studies) and inner setting (19 studies), while factors in the outer setting were least reported (12 studies). Twenty-five factors were reported by at least five studies (25%) and therefore were considered the most prominent ones. Among these factors, we distinguished seven factors as predominant barriers and seven as predominant facilitators. The remaining 11 factors showed a nearly equivalent direction of influence as impeding and facilitating (i.e., indistinguishable). Here we use “predominant” when a factor was clearly and more frequently reported as either a barrier or facilitator, judged by whether at least two thirds of the total number of studies reporting this factor reported it as a barrier or facilitator. Nevertheless, this does not directly imply that these factors are the most important, but it conveys that they are very likely to affect the implementation of TC innovations in either direction of influence. The main findings describing the most prominent factors are presented below, and Fig. 3 provides an overall summary.

### Factors—predominantly barriers

#### Targeted groups

A mismatch between the TC innovation components and the intended profile of the recipients, older persons, was evident to affect its implementation as indicated in nine studies [14, 18, 31, 32, 34–36, 43, 47]. Five studies reported that unclear eligibility criteria of the TC innovation often impeded the identification of older persons that could benefit from it [14, 18, 32, 35, 47]. Another four studies stated that TC innovations were unable to meet the specific care needs of the targeted older persons due to the high frailty and complex conditions of the recipients, confirming an incompatible fit [31, 34, 36, 43].

#### Complexity

The intricacy of the TC innovation design and the difficulty of putting it into action were reported mutually in five studies [14, 31, 36, 38, 39]. Two studies cited that the necessity to involve multiple homecare service providers [14] and informal caregivers [31], and the absence of bundled care payment methods [14] led to difficulty in implementing TC innovations in home settings. Healthcare providers perceived that TC innovations with complex and extensive processes [39], unstandardized or detailed protocols [36], and hard to understand and use tools and checklists [38] affected the implementation negatively.

#### Readiness for implementation: available resources

Low staffing levels [43, 44, 46] and a lack of dedicated staff [14] were common impeding factors to the implementation of TC innovations. Similarly, staff turnover [38, 47, 49] plus losing key team members [39] and major program staff and contact persons [40] affected the implementation negatively. This led to increased costs and weakened relationships between organizations involved in implementing a TC innovation [40]. Heavy workloads [38, 47, 49], time constraints [39, 46], and work schedule pressure [46] also hindered implementation and sometimes led to less staff engagement [38]. Limited availability of needed resources such as equipment and care service provisions [18], as well as financial constraints [47] or a lack of funding [37, 40], were notable barriers to implementation. Moreover, three studies indicated that an inadequate training and education offered to providers and staff hindered their ability to implement new TC innovations [36, 42, 47].

#### Continuity

A disrupted information flow, communication, or relationship between multiple healthcare providers and across organizations was described as cumbersome and impeding to the implementation of TC innovations [14, 18, 32, 34, 42, 46]. Four studies reported that an insufficient, inconsistent, or discontinuous patient information exchange between different organizations often led to delays in coordination of services and care planning, which was the essence of some TC innovations [14, 18, 34, 42]. Furthermore, the inefficient communication and difficulties in maintaining steady working relationships among the TC innovation program staff and, for example, the hospital or nursing home staff were barriers to the implementation [32, 46].

#### Implementation climate: relative priority

The existence of multiple quality improvement initiatives and projects within the organization often hindered the efforts to implement new TC innovations concurrently [33]. Moreover, alternate quality improvement projects posed competition to the introduction of new TC innovations [39] and sometimes a mix of confusion and doubts among the staff on their need [47]. Overall, staff described change fatigue as a main barrier to endorsing new transitional care activities, as well as leadership sometimes failing to actively endorse new transitional care programs [32, 33]. Two studies indicated that major organizational changes also created different priorities among staff and a reduced capacity and motivation to implement new TC innovations [38, 49].

#### Knowledge and beliefs about the intervention

The older persons’ misconceptions about the TC innovation together with a limited awareness of its specific services and goals, as well as a low perceived value, affected the enrolment process and implementation [31, 33, 36]. Moreover, some older persons expressed privacy concerns over aspects of the innovations, such as home visits by care providers, and hence viewed it as a disruption with a little value [33, 35, 36]. Similarly, mixed knowledge and beliefs surrounding the innovation [38], confusion on the innovation’s direction [36], and not knowing what is expected [44, 47] by healthcare providers were reported as hindrances to the implementation. One study cited that care home staff believed that the new intervention would make them highly liable and accountable [45], whereas in another study, staff saw that a mind shift is required or implementation is impeded [46].

#### Planning

Two studies indicated that following a less organized implementation plan with a low-quality and feasibility vision impeded the execution of a TC innovation [33, 39].While another four studies cited that the lack of clear initiation workflows and specific protocols [14, 47], as well as an absence of early induction and explanation of the innovation [35, 44], led to early missteps and confusion in rolling out the TC innovations [35, 47].

### Factors—predominantly facilitators

#### Relative advantage

Four studies reported that the benefits and usefulness offered by a TC innovation facilitated its implementation [35, 38, 44, 46]. Healthcare providers stated that TC innovations with certain supportive tools, such as compiling an older person’s information during transitions between settings, helped staff work more efficiently and thereafter enhanced the implementation [44]. In addition, an improved quality of information transfer and communication between community and nursing home settings offered by one TC innovation’s features was perceived as advantageous by staff [46]. Moreover, the implementation of a TC innovation was facilitated when managers observed incremental benefits such as improved healthcare staff practice and skills [38].

#### Evidence strength and quality

Proven effectiveness and solid evidence on the TC innovation’s ability to ensure positive outcomes enabled its implementation [35, 39, 40]. Outcomes such as low readmission rates [40] and better patient satisfaction [39] resulting from a TC innovation led to a high buy-in from the healthcare providers [40] and a convinced leadership [35], which consequently supported the implementation.

#### Information technology (IT) and health information technology (HIT) resources: HIT systems

The presence of supportive electronic health information systems enhanced the implementation of TC innovations by enabling better communication, shared information documentation, and patient care management across settings [18, 36, 38, 48]. Notably, the incorporation in patients’ electronic files of either a TC innovation-specific checklist [38] or signaling the involvement of a TC manager in the care management [18] facilitated the adoption.

#### Role

Defining clear roles and responsibilities for the key TC innovation implementing team members facilitated the implementation [35]. Three studies reported that key staff played a critical role in implementation, through adhering to the application of the innovation’s specific activities [31, 32], providing regular support, and serving as a liaison and communication channel between different care settings and caregivers [47].

#### Transition roles: frontline staff

Five studies reported that the presence of frontline staff with a designated transition role facilitated the execution of a TC innovation [31, 35, 42, 44, 47]. A role directly attached to the innovation, such as transition care staff [42], advanced practice nurses [31], or a clinical nurse consultant [44], was vital to implement the core components of the innovation by being in direct contact with older persons and able to identify and manage their transition care needs.

#### Reflecting and evaluating—measurement capability and data availability

Regular communication and feedback between staff on the progress of implementing TC innovations, such as sharing successful outcome measures, fostered more leadership support for continuing the implementation [38–40]. Furthermore, ensuring a continuous monitoring of the innovation’s effectiveness and overall performance as well as quality and safety for patients allowed for timely adaptations in the implementation process, together with maintaining its continuity [14, 35, 38].

### Factors—indistinguishable barriers/facilitators

Eleven factors across four domains were highly reported, however with an overall nearly equivalent influence as both impeding and facilitating the implementation of a TC innovation.

#### Cosmopolitanism

Although five studies reported that pre-existing partnerships, the establishment of new external networks, or sharing of practices between various healthcare organizations enabled a faster and better implementation of TC innovations [14, 40–42, 46], four studies indicated poor interorganizational relationships and unwillingness to collaborate as evident barriers [33, 40, 45, 48].

#### External policy and incentives

The presence of external unsupportive laws and regulations, as well as the discontinuity of national funding schemes, showed a negative influence on the implementation of TC innovations in four studies [14, 35, 40, 46]. Conversely, another four studies cited that favorable extrinsic legislative changes [41, 49] or the availability of governmental sponsorship for new TC innovations were facilitators [36, 42].

#### Networks and communications

A challenging team formation with an absence of regular, effective, and clear communication among the members impeded the implementation, as cited in seven studies [14, 32, 34, 43, 46–48]. In contrast, suggested facilitators included established interdisciplinary teams [39], strong coordination [33], or cooperative working relationships across team members [35, 36, 48].

#### Culture

Progressive [33], innovative [32], flexible [40], or problem-solving [35, 49] organizational norms and values with emphasis on patient-centered care [32] fostered implementing new TC innovations. In contrast, a mismatch in cultures between healthcare organizations or the presence of traditional and resistant to change values was shown to hinder the implementation [35, 43, 46, 47, 49].

#### Readiness for implementation (leadership engagement; access to knowledge and information)

Insufficient involvement and a limited support from existing leadership along with a lack of interest in implementing a new TC innovation affected the process negatively [32, 37, 39, 43, 47]. Likewise, failing to provide the required information and initial training to staff on a new TC innovation hindered its implementation [14, 37, 44]. In contrast, a high organizational commitment and sustained leadership [35, 38, 41, 49], and ensuring the access to knowledge and mentoring on the TC innovation, facilitated the implementation [35, 38, 42].

#### Skills, competencies, and other personal attributes

Six studies indicated that a lack of staff expertise, knowledge capacity, and skills, along with insufficient educational levels, often delayed or ultimately hindered the implementation of TC innovations [14, 36, 38, 43, 45, 46]. Conversely, another six studies suggested that staff with a wide experience in long-term care and possessing clinical and technical skills [31, 32, 35, 44, 47, 48], as well as high critical attributes [47], were a great source of implementation facilitation. Similarly, low motivation levels and frustration among the staff [36, 38] or patient’s poor health literacy [34] and no acknowledgement of care needs [31] impeded implementation; yet a high motivation for change had a positive influence [31, 46, 49].

#### Engaging: key stakeholders, innovation participants, organizations, and external context

The challenge to involve actively and early on the key healthcare professionals, patients, family, and external providers in addition to low levels of training and induction activities impeded the implementation of various TC innovations [14, 31, 33, 39–45]. However, a continuous engagement of healthcare providers [36] and the patient [39, 44], alongside stimulating external collaborations [46] or ensuring family inclusion in care goals setting [42], fostered the implementation. Similarly, exercising team-building efforts [14, 39], gaining an early buy-in and support from key staff [32, 38, 48], and advertising the TC innovation well [35] were essential facilitators.

### Perspectives of older persons, family, or informal caregivers on TC innovations

Six studies reported on the overall perception of the older persons and/or their caregivers regarding the transitional care innovation being implemented. Often the feedback was not specific to the implementation aspect, but rather on the innovation’s components, benefits, and satisfaction. Some components of the TC innovations, such as medication management, were perceived as a challenge for patients [37], whereas a transition role, such as a care coordinator [36], clinical nurse consultant [44], or community psychiatric nurse [46], was perceived as highly valuable and beneficial. In addition, the provision of clear information and expectations from the TC innovation was seen as highly satisfactory [44, 47]. Three studies reported that older persons and their caregivers had a mixed experience with the innovation as either satisfying or devaluing, thus sometimes feeling that the components do not fit or meet their care transition needs or wishes [36, 41, 44].

## Discussion

Our study identified an interplay of 25 main factors that acted as barriers and facilitators during the implementation of diverse transitional care innovations. Fourteen factors presented with a predominant direction of influence. The important barriers were linked to the organization’s implementation readiness and climate, targeted older populations, process planning, and users’ knowledge. The significant enabling factors were the innovation’s high relative advantage, transition roles of professionals, and evaluation of the implementation process. Furthermore, we could not distinguish a clear-cut direction for the influence of other key factors. By large, the current findings are in line with previous research and theories suggesting that a range of interrelated factors existing at multiple levels determine the success of the implementation of innovations [50, 51].

Our results indicate that certain factors related to the implementation process and intervention characteristics appear to be specific to transitional care innovations. While the roles of middle managers [52, 53] and champions [54, 55] were indicated as facilitators to implementing general healthcare or long-term care (LTC) innovations, transition roles of frontline staff in LTC were key in facilitating the adoption and execution of TC innovations. Moreover, awareness of existing barriers in designing and tailoring TC innovations to the target population was seen as lacking across many of the studies we reviewed. This could be explained by the specific profile and care transition needs of older persons that seem to be overlooked when developing innovations. Even though the components of some TC innovations entailed the involvement of both older persons and caregivers in the development of care plans, a mismatch of needs occurred. As presented elsewhere, it is highly important to ensure patient engagement in codesigning processes or evaluations of care improvement initiatives such as TC innovations [56, 57]. Moreover and in our attempt to answer the second research question, this review found only few studies that took the perspectives of transitional care recipients into account, while examining the implementation of TC innovations. The role of the older persons and thereby the consideration of their wishes and needs in the implementation process appear to be limited. Hence, the older persons and/or their informal or family caregivers’ reflection on the actual implementation challenges are understudied, since the providers’ perspectives are often those sought after.

Furthermore, the specific context and characteristics of LTC organizations play an integral role in implementing innovations [58–62]. Correspondingly, our results indicated that the LTC organizational culture, implementation climate, readiness for implementation, implementation process, the individuals’ skills and attributes, and internal communication dynamics have a major impact on the uptake of several TC innovations. This provides further evidence regarding the theory on organizational readiness for change (ORC) by Weiner [63], which explains that fostering the organization’s capacity, commitment, and efficacy to change are notable drivers in creating readiness and ultimately enhance implementation. Similarly, our results affirm the work of Attieh et al. [64], in which five core theoretical components of ORC were identified including the organizational dynamics, change process, innovation readiness, institutional readiness, and personal readiness. Our results indicate that lacking resources often hindered the implementation of various TC innovations, and that the organizational culture had a prominent yet mixed influence on bringing about a change. According to Weiner [63], organizational resources and culture are among the contextual factors that can affect the organizational capacity and readiness for change. This review also identified that the individuals’ skills, knowledge, perceived attitudes, and designated roles were prominent factors in implementing an innovation. This is evident as per Holt’s et al. [65] and Weiner’s [63] concepts of change efficacy, which explain that individuals in an organization with a high shared collective capability and confidence to implement new tasks successfully can enhance the organizational readiness for change. In addition, our findings on the importance of implementation climate explained by the individuals’ relative priority to implement a TC innovation within an organization as well as their motivation levels relate to the organizational change commitment [63, 65, 66]. Lastly, the literature indicated that organizational leadership and internal communication dynamics are instrumental in generating readiness for change, as was mirrored in our results [63, 66].

## Future recommendations

### Research

Prospective studies on the degree of influence of each identified barrier and facilitator on the implementation of a TC innovation are needed. This will enable the development of tailored implementation strategies by addressing the prioritized factors. Furthermore, focusing on the older person’s perspective when studying the implementation process of TC innovations is required. This will alleviate the discontinuous and problematic care transitions for the older population.

### Policy and practice in transitional care

Future implementation of TC innovations can benefit from a preassessment of the key components that underpin an LTC organization’s readiness for change by using established ORC measurement instruments [67]. Overall, these measures can offer an initial support for LTC organizations to better prepare for implementing innovations by reducing blinded change efforts. Simultaneously, LTC organizations can leverage their readiness for implementing change by, for example, adopting the concept of innovation management as reflected in A.T. Kearney’s House of Innovation [68]. This framework invites organizations to start with an innovation strategy and build an innovative and open culture. In addition, organizations must manage the innovation’s process in an integrated and continuous manner from idea conception to implementation, as a way to avoid inefficiencies and ensure timely positive outcomes. Bates et al. [58] emphasized the power to create successful innovative healthcare environments by making innovation a strategic priority. Henceforth, we recommend LTC organizations bolster their innovation readiness and management, whereby they encourage among professionals an incessant mindset of “change is the norm.” Nevertheless, this readiness should be fostered across the continuum of care spanning multiple LTC settings, given the nature of TC. In addition, transition roles or implementation support practitioners [69] should be instituted to better operationalize innovations in TC.

## Strengths and limitations

We consider the combined use of CFIR and CTF a methodological asset for conducting this review, especially in the process of data extraction and mapping of factors. The CFIR provided an intricate yet systematic way to understand the interconnectedness of the numerous factors. The inclusion of constructs from the CTF was found vital in detecting factors specific to care transitions. On the other hand, we acknowledge that different or additional factors could have been found had we chosen to use another framework.

This review has some limitations. First, it is subject to publication bias, since we only included articles published in peer-reviewed journals and excluded gray literature, preregistries, and policy documents. Second, even though we used an extensive search strategy to identify relevant studies on implementing TC innovations, we might have missed some potentially relevant papers, as the aim of innovations in LTC is not always clearly described. Third, not all records were screened by two persons; only a random selection of 10% of the initial total records was screened by a second reviewer for titles and abstracts. Though agreement seemed satisfactory, we cannot fully rule out that some relevant sources could have been missed. Fourth, we did not perform critical appraisal for the included studies, even though it is not mandatory in scoping reviews’ methodology, it could have added to the interpretability of the findings.

## Conclusions

A diversity of factors impact the implementation of TC innovations; these include the innovation’s complexity, relative advantage and evidence strength, organizational readiness for implementation, individuals’ knowledge and beliefs, and the implementation process planning and evaluation. To ensure implementation potential, TC innovations need to address the right older target population, and transition roles for staff should be developed as key steps. LTC organizations can benefit from collaborating and leveraging concurrently their readiness for change along with adopting innovation management in order to succeed in implementing TC innovations. Furthermore, minimizing the confusion around how implementing innovation works holds the potential to improve care transitions for older persons.

## Supplementary Information

**Additional file 1.** PRISMA-ScR checklist. This file describes a checklist of items that are covered and reported in this scoping review as per the PRISMA-ScR.

**Additional file 2.** Search strategy for electronic databases. Microsoft Word Document (.docx). This file includes the search strategy for the databases PubMed/MEDLINE, EMBASE, & CINAHL.

**Additional file 3.** Consolidated Framework for Implementation Research & Care Transitions Framework constructs description. This file provides the description and definitions of the CFIR constructs and the constructs selected and used from the CTF.

## Data Availability

Data generated and analyzed during this study are avaliable from the corresponding author on reasonable request.

## Abbreviations

TC: Transitional care
LTC: Long-term care
PRISMA-ScR: Preferred Reporting Items for Systematic Reviews and Meta-Analyses extension for Scoping Reviews
PRISMA: Preferred Reporting Items for Systematic Reviews and Meta-analyses
CFIR: Consolidated Framework for Implementation Research
CTF: Care Transitions Framework
IT: Information technology
HIT: Health information technology
ORC: Organizational readiness for change
