# Supplementary material for: A myriad of factors influencing the implementation of transitional care innovations: a scoping review
Source: Implement Sci. 2021 Feb 26;16:21. doi: 10.1186/s13012-021-01087-2 (PMC7912549; doi:10.1186/s13012-021-01087-2)
Supplement: Supplementary file 2 — Additional file 2. Search strategy for electronic databases. Microsoft Word Document (.docx). This file includes the search strategy for the databases PubMed/MEDLINE, EMBASE, & CINAHL. [file 13012_2021_1087_MOESM2_ESM.docx]

**Additional file 2: Search strategy for the electronic databases**

**PubMed/MEDLINE**

**Concept 1: Implementation**

| MeSH terms | Keywords |
| --- | --- |
| Implementation Science[MeSH] | implement*[tiab] OR adopt*[tiab] OR integrat*[tiab] OR disseminat*[tiab] OR promot*[tiab] |

**Concept 2: Innovation**

| MeSH terms | Keywords |
| --- | --- |
| Diffusion of Innovation[MeSH] OR Organizational Innovation[MeSH] OR Inventions[MeSH] OR Change Management[MeSH] | program*[tiab] OR model*[tiab] OR intervention*[tiab] OR system*[tiab] OR practice*[tiab] OR tool*[tiab] OR approach*[tiab] OR pathway*[tiab] OR change*[tiab] OR innovat*[tiab] OR invention*[tiab] |

**Concept 3: Care Transition**

| MeSH terms | Keywords |
| --- | --- |
| Patient Transfer[MeSH] OR Transitional Care[MeSH] OR Patient Handoff[MeSH] | care transition*[tiab] OR "transition of care"[tiab] OR "transitions of care"[tiab] OR  care transfer*[tiab] OR "transfer of care"[tiab] OR  patient transition*[tiab] OR patient transfer*[tiab] OR "transfer of patient"[tiab] OR  patient relocat*[tiab] OR  patient handover[tiab] OR patient hand-over[tiab] OR patient handoff*[tiab] |

**Concept 4: Older Persons**

| MeSH terms | Keywords |
| --- | --- |
| Aged[MeSH] OR Geriatrics[MeSH] OR Frail Elderly[MeSH] | older*[tiab] OR elder*[tiab] OR frail*[tiab] OR geriatri*[tiab] OR old age*[tiab] OR oldest old*[tiab] OR senior*[tiab] OR very old*[tiab] OR older people[tiab] OR older patient*[tiab] OR older age*[tiab] OR older adult*[tiab] OR older population*[tiab] OR older person*[tiab] OR geriatric*[tiab] |

**EMBASE**

**Concept 1: Implementation**

| Subject Headings | Keywords |
| --- | --- |
| *----* | (implement* OR promot* OR adopt* OR integrat* OR disseminat*).ti,ab,kw. |

**Concept 2: Innovation**

| Subject Headings | Keywords |
| --- | --- |
| exp Organization/ OR | (program* OR model* OR intervention* OR system* OR practice* OR tool* OR approach* OR pathway* OR change* OR innovat* OR invention*).ti,ab,kw. |

**Concept 3: Care transition**

| Subject Headings | Keywords |
| --- | --- |
| *----* | (care transition* OR "transition of care" OR "transitions of care" OR care transfer* OR "transfer of care" OR patient transition* OR patient transfer* OR "transfer of patient" OR patient relocat* OR patient handover OR patient hand-over OR patient handoff*).ti,ab,kw. |

**Concept 4: Older persons**

| Subject Headings | Keywords |
| --- | --- |
| exp Aged/ OR | (older* OR elder* OR frail* OR geriatri* OR old age* OR oldest old* OR senior* OR very old* OR older people OR older patient* OR older age* OR older adult* OR older population* OR older person* OR geriatric*).ti,ab,kw. |

**CINAHL**

**Concept 1: Implementation**

| Subject Headings | Keywords |
| --- | --- |
| (MH "Implementation Science") | TI implement* OR TI promot* OR TI adopt* OR TI integrat* OR TI disseminat* OR  AB implement* OR AB promot* OR AB adopt* OR AB integrat* AB disseminat* |

**Concept 2: Innovation**

| Subject Headings | Keywords |
| --- | --- |
| (MH "Diffusion of Innovation+") | TI innovat* OR TI change* OR TI invention* OR TI model* OR TI program* OR TI intervention* OR TI system* OR TI practice* OR TI tool* OR TI approach* OR TI pathway* OR  AB innovat* OR AB change* OR AB invention* OR AB model* OR AB program* OR AB intervention* OR AB system* OR AB practice* OR AB tool* OR AB approach* OR AB pathway* |

**Concept 3: Care Transition**

| Subject Headings | Keywords |
| --- | --- |
| (MH "Transitional Care") | TI "care transition*" OR TI "transition of care" OR TI "transitions of care" OR TI "care transfer*" OR TI "transfer of care" OR TI "patient transition*" OR TI "patient transfer*" OR TI "transfer of patient" OR TI "patient relocat*" OR TI "patient handover" OR TI "patient hand-over" OR TI "patient handoff*" OR  AB "care transition*" OR AB "transition of care" OR AB "transitions of care" OR AB "care transfer*" OR AB "transfer of care" OR AB "patient transition*" OR  AB "patient transfer*" OR AB "transfer of patient" OR AB "patient relocat*" OR AB "patient handover" OR AB "patient hand-over" OR AB "patient handoff*" |

**Concept 4: Older persons**

| Subject Headings | Keywords |
| --- | --- |
| (MH "Aged+") | TI elder* OR TI older* OR TI frail* OR TI geriatri* OR TI old age* OR TI oldest old* OR TI senior* OR TI very old* OR TI older people OR TI older patient* OR TI older age* OR TI older adult* OR TI older population* OR TI older person* OR TI geriatric* OR  AB elder* OR AB older* OR AB frail* OR AB geriatri* OR AB old age* OR AB oldest old* OR AB senior* OR AB very old* OR AB older people OR AB older patient* OR AB older age* OR AB older adult* OR AB older population* OR AB older person* OR AB geriatric* |
