# Supplementary material for: A myriad of factors influencing the implementation of transitional care innovations: a scoping review
Source: Implement Sci. 2021 Feb 26;16:21. doi: 10.1186/s13012-021-01087-2 (PMC7912549; doi:10.1186/s13012-021-01087-2)
Supplement: Supplementary file 3 — Additional file 3. Consolidated Framework for Implementation Research & Care Transitions Framework constructs description. This file provides the description and definitions of the CFIR constructs and the constructs selected and used from the CTF. [file 13012_2021_1087_MOESM3_ESM.pdf]

Additional file 3: Consolidated Framework for Implementation Research (CFIR) & Care Transitions Framework (CTF) constructs description

CFIR constructs / CTF selected constructs		Short Description
**I. Domain: INTERVENTION CHARACTERISTICS**		
**A**	**Intervention Source**	Perception of key stakeholders about whether the intervention is externally or internally developed.
**B**	**Evidence Strength & Quality**	Stakeholders' perceptions of the quality and validity of evidence supporting the belief that the intervention will have desired outcomes.
**C**	**Relative Advantage**	Stakeholders' perception of the advantage of implementing the intervention versus an alternative solution.
**D**	**Adaptability**	The degree to which an intervention can be adapted, tailored, refined, or reinvented to meet local needs.
**E**	**Trialability**	The ability to test the intervention on a small scale in the organization, and to be able to reverse course (undo implementation) if warranted.
**F**	**Complexity**	Perceived difficulty of implementation, reflected by duration, scope, radicalness, disruptiveness, centrality, and intricacy and number of steps required to implement.
**G**	**Design Quality & Packaging**	Perceived excellence in how the intervention is bundled, presented, and assembled.
**H**	**Cost**	Costs of the intervention and costs associated with implementing the intervention including investment, supply, and opportunity costs.
**CTF**	**Vision & Change Strategy**	The proposed changes envisioned by the intervention and the theory of change: how the intervention is supposed to work, what it is meant to achieve or do. May be explicated in logic models, goals, outcomes, performance measures.
**CTF**	**Targeted Groups**	Staff and others (vendors, patients) who are the intended recipients or beneficiaries of the intervention.
**CTF**	**Feasibility**	Target group and other stakeholders' perceptions of the extent to which the intervention can be successfully used or carried out within the organization(s).
**CTF**	**Compatibility**	Target group and stakeholder perception of the alignment of the meaning, values, and norms attached to care transitions with those held by members of the organization(s).
**CTF**	**Radicaliness**	Target group and other stakeholder perceptions of the degree of difference between the change envisioned and the current state of care transitions
**CTF**	**User Control**	The degree to which the intervention relies on the end-users' authority/skill to implement the intervention on their own vs. reliance on experts.

**CTF**	**Location of intervention activity**	Components of the intervention conducted outside the hospital/clinic/office setting using external service providers and organizations.
**CTF**	**Workflows**	Tasks and workflows, including interdependencies between them that are the focus of the intervention or will be affected by it.
**CTF**	**Task/Process standardization**	Degree to which the intervention seeks to standardize tasks and processes that require iterative consultation.
**CTF**	**History**	Experiences with similar interventions within the organizations or within the target groups.
**II. Domain: OUTER SETTING**		
**A**	**Patient Needs & Resources**	The extent to which patient needs, as well as barriers and facilitators to meet those needs, are accurately known and prioritized by the organization.
**B**	**Cosmopolitanism**	The degree to which an organization is networked with other external organizations.
**C**	**Peer Pressure**	Mimetic or competitive pressure to implement an intervention; typically because most or other key peer or competing organizations have already implemented or are in a bid for a competitive edge.
**D**	**External Policy & Incentives**	A broad construct that includes external strategies to spread interventions, including policy and regulations (governmental or other central entity), external mandates, recommendations and guidelines, pay-for-performance, collaboratives, and public or benchmark reporting.
**CTF**	**Technological Environment**	The technological trends and movements and the availability of technological innovations that may affect the intervention and its context.
**CTF**	**Population Needs and Resources**	Prevalence of conditions and disease in the population served and the characteristics of the community that are determinants of health status.
**CTF**	**Community Resources**	Availability and access of service providers, aging resources, and multiple levels of community services and supports not directly involved in the intervention.
**III. Domain: INNER SETTING**		
**A**	**Structural Characteristics**	The social architecture, age, maturity, and size of an organization.
**B**	**Networks & Communications**	The nature and quality of webs of social networks and the nature and quality of formal and informal communications within an organization.
**C**	**Culture**	Norms, values, and basic assumptions of a given organization.
**D**	**Implementation Climate:**	The absorptive capacity for change, shared receptivity of involved individuals to an intervention, and the extent to which use of that intervention will be rewarded, supported, and expected within their organization.

**D.1**	***Tension for Change***	The degree to which stakeholders perceive the current situation as intolerable or needing change.
**D.2**	***Compatibility***	The degree of tangible fit between meaning and values attached to the intervention by involved individuals, how those align with individuals' own norms, values, and perceived risks and needs, and how the intervention fits with existing workflows and systems.
**D.3**	***Relative Priority***	Individuals' shared perception of the importance of the implementation within the organization.
**D.4**	***Organizational Incentives & Rewards***	Extrinsic incentives such as goal-sharing awards, performance reviews, promotions, and raises in salary, and less tangible incentives such as increased stature or respect.
**D.5**	***Goals and Feedback***	The degree to which goals are clearly communicated, acted upon, and fed back to staff, and alignment of that feedback with goals.
**D.6**	***Learning Climate***	A climate in which: a) leaders express their own fallibility and need for team members' assistance and input; b) team members feel that they are essential, valued, and knowledgeable partners in the change process; c) individuals feel psychologically safe to try new methods; and d) there is sufficient time and space for reflective thinking and evaluation.
**CTF**	***Mandate***	Whether compliance with the intervention is expected within the organization.
**CTF**	***Accountability***	Whether entities are subject to tangible consequences for noncompliance.
**E**	**Readiness for Implementation:**	Tangible and immediate indicators of organizational commitment to its decision to implement an intervention.
**E.1**	***Leadership Engagement***	Commitment, involvement, and accountability of leaders and managers with the implementation.
**E.2**	***Available Resources***	The level of resources dedicated for implementation and on-going operations, including money, training, education, physical space, and time.
**E.3**	***Access to Knowledge & Information***	Ease of access to digestible information and knowledge about the intervention and how to incorporate it into work tasks.
**CTF**	***Staff Commitment***	The degree of clinician, transitional, and community care staff, patient, and caregiver involvement in transition planning
**CTF**	**IT and HIT Resources:**	Technological infrastructure in place to support electronic information management, including IT that crosses organizations.
**CTF**	***HIT Systems***	Electronic information management infrastructure and technologies available to clinicians to manage patient care, data, and communications.
**CTF**	***IT Systems***	Technological systems and capabilities to support care transitions.

**CTF**	***HIT/IT Accessibility***	Includes features of the physical, technical, and social environment in the organization that determine the use, accessibility, and acceptability of technology in patient care.
**CTF**	**Other Resources**	Resources for implementation and ongoing operations to support change and innovation, including grant or other funding specific to care transitions.
**CTF**	**Patient Self-management Infrastructure**	Training, counseling, and education available to patients prior to the intervention within the hospital and ambulatory setting.
**CTF**	**Continuity**	Information continuity (exchange of information) and relationship continuity, both with providers and patients/caregivers and across organizations.
**CTF**	**Patient/caregiver-centeredness**	Extent to which the organization(s) knows and prioritizes patient and caregiver goals, needs, and preferences, and has the resources and services to meet them
**IV. Domain: CHARACTERISTICS OF INDIVIDUALS**		
**A**	**Knowledge & Beliefs about the Intervention**	Individuals' attitudes toward and value placed on the intervention as well as familiarity with facts, truths, and principles related to the intervention.
**B**	**Self-efficacy**	Individual belief in their own capabilities to execute courses of action to achieve implementation goals.
**C**	**Individual Stage of Change**	Characterization of the phase an individual is in, as he or she progresses toward skilled, enthusiastic, and sustained use of the intervention.
**D**	**Individual Identification with Organization**	A broad construct related to how individuals perceive the organization, and their relationship and degree of commitment with that organization.
**E**	**Other Personal Attributes**	A broad construct to include other personal traits such as tolerance of ambiguity, intellectual ability, motivation, values, competence, capacity, and learning style.
**CTF**	**Collective Efficacy**	Conviction of individuals and teams involved that the intervention can be carried out in cooperation with each other.
**CTF**	**Skills and Competencies**	Degree of relevant subject matter expertise, skills, and competencies within the implementing team, unit, and organization.
**CTF**	**Role**	Individual's role and responsibility for the intervention. The degree of multiple or shared roles.
**CTF**	**Authority**	Individual provider's perceived and actual degree of authority to make decisions and act autonomously.
**CTF**	**Socioeconomic Demographics**	Characteristics related to the individual's socioeconomic status.
**CTF**	**Patient Needs and Resources**	Patient priorities for health and health care priorities and the social and economic capital to address those priorities.
**CTF**	**Caregiver Needs and Resources**	Caregiver priorities for health and health care, and the social and economic capital to address those priorities.

V. Domain: PROCESS		
**A**	**Planning:**	The degree to which a scheme or method of behavior and tasks for implementing an intervention are developed in advance, and the quality of those schemes or methods.
**CTF**	***Assessing***	Formal assessment of care transitions issues; the needs of the users; barriers to change; the timing of these activities relative to implementation.
**CTF**	***Contingency Planning***	Plans for adaptation in response to various scenarios and outcomes.
**CTF**	**Acquiring and Allocating Resources**	Resources dedicated to implementing the intervention; the adequacy of those allocations.
**CTF**	**Process Ownership**	The diversity of transition roles involved in processes of implementation; authority and accountability for these activities.
**CTF**	**Transition Roles:**	Roles of individuals involved in the decision to adopt, execute, and facilitate the intervention.
**CTF**	***Organizational Leaders***	Managers and others with the authority to dedicate resources and make decisions to adopt, maintain, or abandon the implementation.
**CTF**	***Frontline Staff***	Administrative staff, providers (within and outside the organization) who will carry out the intervention or be affected by it.
**CTF**	***Integrators***	Individuals who build relationships between organizations and create linkages to facilitate the intervention.
**CTF**	***Patients, Caregivers, and Other Stakeholders***	Patient and his/her family members, and members of the family's support network.
**B**	**Engaging:**	Attracting and involving appropriate individuals in the implementation and use of the intervention through a combined strategy of social marketing, education, role modeling, training, and other similar activities.
**B.1**	***Opinion Leaders***	Individuals in an organization who have formal or informal influence on the attitudes and beliefs of their colleagues with respect to implementing the intervention.
**B.2**	***Formally Appointed Internal Implementation Leaders***	Individuals from within the organization who have been formally appointed with responsibility for implementing an intervention as coordinator, project manager, team leader, or other similar role.
**B.3**	***Champions***	"Individuals who dedicate themselves to supporting, marketing, and 'driving through' an [implementation]", overcoming indifference or resistance that the intervention may provoke in an organization.
**B.4**	***External Change Agents***	Individuals who are affiliated with an outside entity who formally influence or facilitate intervention decisions in a desirable direction.

**B.5**	***Key Stakeholders****	Individuals from within the organization that are directly impacted by the innovation, e.g., staff responsible for making referrals to a new program or using a new work process.
**B.6**	***Innovation Participants****	Individuals served by the organization that participate in the innovation, e.g., patients in a prevention program in a hospital.
**CTF**	***Engaging Organizations, External Context***	Developing and capitalizing on relationships with providers, leaders, and frontline staff in the implementing organizations, and to external providers, resources, funders.
**C**	**Executing:**	Carrying out or accomplishing the implementation according to plan.
**CTF**	***Decisionmaking***	Frequency, duration, and timing of the activities involved in making decisions. The directionality of these activities.
**CTF**	***Staging and Iteration***	Degree to which the care transition is carried out in iterative, incremental steps or implemented in its entirety within a specified period.
**D**	**Reflecting & Evaluating:**	Quantitative and qualitative feedback about the progress and quality of implementation accompanied with regular personal and team debriefing about progress and experience.
**CTF**	***Measurement Capability and Data Availability***	Availability of timely data. Capacity for monitoring, evaluation, and process improvement. Includes measurement differences; accountability for collection, documentation, and analysis.

*Two additional constructs (engaging: key stakeholders, innovation participants) under engaging in the process domain were added as per CFIR research group (https://cfirguide.org/).

References:

1) Damschroder LJ, Aron DC, Keith RE, Kirsh SR, Alexander JA, Lowery JC. Fostering implementation of health services research findings into practice: a consolidated framework for advancing implementation science. Implement Sci. 2009;4:50.
2) Dy SM, Ashok M, Wines RC, Rojas Smith L. A framework to guide implementation research for care transitions interventions. J Healthc Qual. 2015;37(1):41-54.
